# A burning question: what are the risks and benefits of mammalian torpor during and after fires?

**DOI:** 10.1093/conphys/coy057

**Published:** 2018-10-11

**Authors:** Fritz Geiser, Clare Stawski, Anna C Doty, Christine E Cooper, Julia Nowack

**Affiliations:** 1 Centre for Behavioural and Physiological Ecology, Zoology, University of New England, Armidale, Australia; 2 Department of Biology, Norwegian University of Science and Technology, Trondheim, Norway; 3 Department of Biological Sciences, Arkansas State University, Jonesboro, AR, USA; 4 School of Molecular and Life Sciences, Curtin University, Perth, Western Australia; 5 School of Natural Sciences and Psychology, Liverpool John Moores University, Byrom Street, Liverpool, UK

**Keywords:** Daily torpor, ecophysiology, foraging, hibernation, mammal, wildfire

## Abstract

Although wildfires are increasing globally, available information on how mammals respond behaviourally and physiologically to fires is scant. Despite a large number of ecological studies, often examining animal diversity and abundance before and after fires, the reasons as to why some species perform better than others remain obscure. We examine how especially small mammals, which generally have high rates of energy expenditure and food requirements, deal with fires and post-fire conditions. We evaluate whether mammalian torpor, characterised by substantial reductions in body temperature, metabolic rate and water loss, plays a functional role in survival of mammals impacted by fires. Importantly, torpor permits small mammals to reduce their activity and foraging, and to survive on limited food. Torpid small mammals (marsupials and bats) can respond to smoke and arouse from torpor, which provides them with the possibility to evade direct exposure to fire, although their response is often slowed when ambient temperature is low. Post-fire conditions increase expression of torpor with a concomitant decrease in activity for free-ranging echidnas and small forest-dwelling marsupials, in response to reduced cover and reduced availability of terrestrial insects. Presence of charcoal and ash increases torpor use by captive small marsupials beyond food restriction alone, likely in anticipation of detrimental post-fire conditions. Interestingly, although volant bats use torpor on every day after fires, they respond by decreasing torpor duration, and increasing activity, perhaps because of the decrease in clutter and increase in foraging opportunities due to an increase in aerial insects. Our summary shows that torpor is an important tool for post-fire survival and, although the physiological and behavioural responses of small mammals to fire are complex, they seem to reflect energetic requirements and mode of foraging. We make recommendations on the conditions during management burns that are least likely to impact heterothermic mammals.

## Introduction

Changes in global weather patterns are predicted to increase the frequency and intensity of severe events such as storms, floods and fires (Diffenbaugh and Field, 2013; IPCC, 2014). Severe wildfires are increasing worldwide and, although in the past these have occurred mainly in the warm season, the recent (2017−18) widely publicised wildfires in Australia and California have occurred in winter. In other regions of the world the timing of the ‘fire season’ has also extended well beyond ‘summer’ (Flannigan *et al.*, 2009). With regard to geography, traditionally fire-prone regions include the Mediterranean and several regions in Africa, California, South America and Australia, but extensive wildfires also have been observed in Canada, China and other parts of the world including north-western Europe.

In Australia and other regions ‘fuel reduction burns’, ‘prescribed fires’ or ‘management burns’, which are usually low-intensity burns, are generally conducted during the cold season in an attempt to reduce the severity of wildfires in the following warm season. The effectiveness of these burns and their impact on ecosystems have been questioned (Whelan, 2002; Fernandes and Botelho, 2003; Boer *et al.*, 2009; Enright and Fontaine, 2014) and this is an ongoing topic of debate, unfortunately mainly in relation to human lives and property. With regard to animals, prescribed burns during the cold season will present different challenges compared with wildfires that usually occur in the warm season.

Despite the acute and direct threat to animal life during both wildfires and management burns, very little is known about how mammals deal with fire, nor how they cope with a denuded post-fire environment where food and shelter are often reduced and predation pressure usually increases (McGregor *et al.*, 2016). Most studies have been ecological in nature and involved pre- and post-fire trapping, or assessing abundance or mortality of animals in another way as, for example, by visual observation of animal numbers or via camera traps (Thompson *et al.*, 1989; Clark and Kaufman, 1990; Letnic *et al.*, 2004; Recher *et al.*, 2009). Although such studies are important as they provide basic information concerning survival and persistence, they cannot reveal the mechanisms by which mammals behaviourally and physiologically respond to fires. Such mechanistic studies require quantification of behavioural and physiological variables pre- and post-fire and can be logistically challenging, especially for wildfires.

We do know that the response of mammals to fire can differ among large mammals, small terrestrial quadrupedal mammals and small volant mammals (bats). Large mammals typically avoid fires, and generally mortality rates are low for large mammals such as ungulates and bears, being ≤1% of populations during a wildfire in Yellowstone National Park, North America (Singer *et al.*, 1989; French and French, 1996). Even medium-sized arboreal mammals such as mountain brush-tail possums (*Trichosurus cunninghami*) can survive the direct impact of fires (Banks *et al.*, 2011). However, not all large mammals survive and especially when fires are hot and extensive they may cause mortality, often from smoke inhalation (Singer *et al.*, 1989). For example, a wildfire resulted in 18% population mortality of African elephants (*Loxodonta africana*), reduced post-fire home range size and increased faecal stress hormones of cows (Woolley *et al.*, 2008). Some, but not all, monitored swamp wallabies (*Wallabia bicolor*) died during and after a wildfire, but all survived a management burn, near Sydney, Australia (Garvey *et al.*, 2010). In a severe wildfire in the Warrumbungle National Park in New South Wales, Australia (2013), a pre-fire overpopulation of grey kangaroos (*Macropus giganteus*) was to a large extent extinguished (Stawski *et al.*, 2014). Although some animals may have escaped to adjacent farm land, the large number of wedge-tail eagles (*Aquila audax*) present after the fire suggests plenty of scavenging opportunities. An influx of scavengers post-fire also occurred in the Yellowstone National Park, where bears, eagles and ravens invaded after fires to feed on carcasses (French and French, 1996). Overall, it appears that in the long-term populations of large mammals survive and are re-established rapidly in post-fire landscapes due to their mobility, which also seems to be the reason why large herbivores show little fear of fire and may graze in close proximity (French and French, 1996). Indeed fire has a direct benefit for some large grazing mammals, for example those which inhabit areas maintained by fire such as grasslands or large open forest gaps, or feed on nutritious post-fire vegetation growth. Fire may also reduce the incidence of parasitism and associated disease by impacting on parasite life-stages associated with vegetation (Pausas and Parr, 2018).

Although small terrestrial mammals also can evade fires by running away (Geluso *et al.*, 1986), usually they cannot outrun fast fires because of their slow speed and high cost of locomotion (Tucker, 1975; Garland *et al.*, 1988). However, their small size allows them to hide in underground burrows, rock crevices or other locations that provide safety from fire (Geluso *et al.*, 1986; Engstrom, 2010; Pausas and Parr, 2018). Many small burrowing rodents employ this strategy as do other small terrestrial mammals such as carnivorous marsupial antechinus (Recher *et al.*, 2009; Stawski *et al.*, 2015a; Matthews *et al.*, 2017). Although many small mammals may survive the direct impact of the fire, some individuals do die from burns, heat, asphyxiation, predation and direct physiological stress during the fire, and fires also result in a decrease in cover and food availability for some time after the actual fire event (Chew *et al.*, 1959; Crowner and Barrett, 1979; Erwin and Stasiak, 1979; Lunney *et al.*, 1987; Simons, 1989; Kaufman *et al.*, 1990; Recher *et al.*, 2009). Consequently, the longer-term limited food and water of a post-fire landscape may present a more severe challenge to small mammals because of their relatively high energy demands and foraging requirements, especially at low ambient temperatures (*T*_a_). Reduction in cover may increase vulnerability to predation, exacerbated by predators invading the area in response to improved hunting conditions (Körtner *et al.*, 2007; Engstrom, 2010; Stawski *et al.*, 2015a; Leahy *et al.*, 2016; McGregor *et al.*, 2016; Hovick *et al.*, 2017; Hradsky *et al.*, 2017). Bats differ from other small terrestrial mammals because of their ability to fly and to move long distances quickly and economically (Tucker, 1975). Bats also have access to both aerial and ground-dwelling prey, so their response to fire may differ to that of other small mammals.

One effective way to deal with the challenge of a fire-denuded landscape and reduced food availability would be to use torpor. Mammalian torpor is likely used by about ¼–½ of all mammals (Geiser and Körtner, 2010), is the most effective energy conservation mechanism available to mammals, is especially common in small mammals including bats and is characterised by pronounced reductions in energy and water requirements even at relatively high *T*_a_ (Macmillen, 1965; Lyman *et al.*, 1982; Boyer and Barnes, 1999; Cooper *et al.*, 2005; Withers and Cooper, 2008; Cory Toussaint *et al.*, 2010; Stawski and Geiser, 2011; Kronfeld-Schor and Dayan, 2013; Johnson and Lacki, 2014; Withers *et al.*, 2016). Torpor can reduce energy expenditure by more than 99% in comparison to normothermia (high and constant body temperature, *T*_b_) and enables some species to survive without food for many months (Geiser, 2007; Hoelzl *et al.*, 2015; Ruf and Geiser, 2015; Nowack *et al.*, 2017). Although once widely considered an adaptation to cold climates, torpor is used in the wild by many mammals living in diverse habitats ranging from the arctic to the tropics (Boyer and Barnes, 1999; McKechnie and Mzilikazi, 2011; Dausmann, 2014; Ruf and Geiser, 2015), and appears particularly important for many small mammals in arid and unpredictable habitats (Lovegrove, 2000; Geiser, 2004; Genin 2008; Munn *et al.*, 2010). Torpor is not only used in winter, but also in summer, for example, in response to drought, inclement weather or reduced food availability and can enable reproduction when resources are limited (Turbill *et al.*, 2003; McKechnie and Mzilikazi, 2011; Stawski and Geiser, 2011; Dzal and Brigham, 2013; Dausmann, 2014; McAllan and Geiser, 2014; Geiser *et al.*, 2017; Nowack *et al.*, 2017). However, the role of mammalian torpor in dealing with the immediate and consequential effects of fires has only recently been investigated.

**Figure 1:**
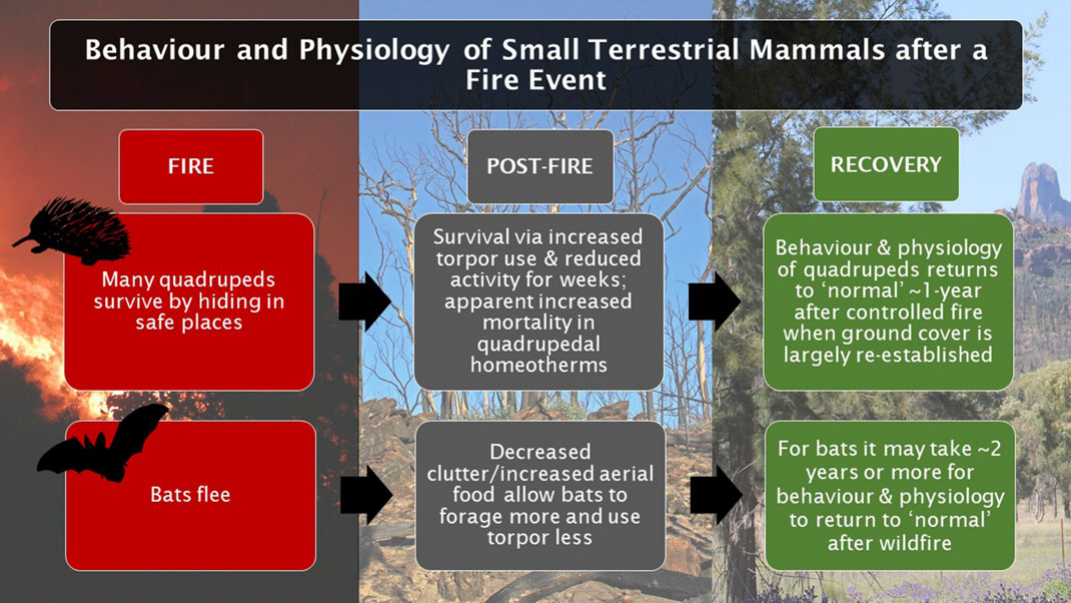
Infographic: The temporal sequence of the biology of small quadrupedal and volant terrestrial mammals during and after a fire. We thank Michael Barritt for the fire picture.

Our review will address what is known about the physiology and behaviour of small mammals during and after fires. We mainly, but not exclusively, report data from the southern hemisphere reflecting the focus of this special issue. The review will focus especially on the advantages and disadvantages of torpor during and after fire, and how torpor is related to foraging behaviour. We will report whether and how small mammals respond to the direct threat of fire by sensing smoke or noise of fire, and how they deal with a post-fire environment. Quadrupedal small mammals and volant bats will be discussed separately with regard to the post-fire responses because their different mode of locomotion and ability to move over large distances presumably impact on their response to fire (Fig. 1).

## Can torpid mammals detect smoke and the noise from fire?

How torpid terrestrial mammals and bats react to fire cues, such as smoke and noise from fire (Table 1) is acutely important, particularly in the context of management burns as these are often conducted during the cold season when many small mammals are likely to be in deep and prolonged torpor. In comparison, most wildfires occur in summer when torpor, if it occurs, tends to be shallow and brief. During torpor, locomotor and sensory capabilities are reduced (Scesny, 2006; Rojas *et al.*, 2012; Luo *et al.*, 2014; Nowack *et al.*, 2016a; Bartonička *et al.*, 2017), which could prevent them from sensing and reacting to smoke and other fire cues in time to escape. However, torpid dunnarts (*Sminthopsis crassicaudata*), pygmy-possums (*Cercartetus nanus*) and bats (*Lasiurus borealis* and *Nyctophilus gouldi*) can respond to smoke, but the response is slowed at low *T*_a_ (Scesny, 2006; Stawski *et al.*, 2015b; Nowack *et al.*, 2016a; Doty *et al.*, 2018). Dunnarts, *S. crassicaudata*, in shallow torpor with a *T*_b_ of ~18 to 25°C rewarmed from torpor about 40 min after smoke exposure. Torpid pygmy-possums, *C. nanus*, at a *T*_a_ of 15°C, responded to smoke after 6–8 min by increasing metabolic rate and aroused or partially aroused within ~30 min, whereas at a *T*_a_ of 10°C and a *T*_b_ of ~13°C only some individuals responded and only one aroused (Nowack *et al.*, 2016a). The response of dormant animals to fire cues is not restricted to mammals; aestivating reed frogs (*Hyperolius nitidulus*) responded to the sound of fires by moving to protective cover (Grafe *et al.*, 2002).

**Table 1:** Physiological and behavioural responses of small mammals to fire

Group/species	Site	Observation	Source
Monotremes			
Echidna *Tachyglossus aculeatus*	Field	Echidnas use torpor during controlled burn, but some die. Post-fire increase in torpor use, activity decreases.	Nowack *et al.* (2016b)
Marsupials			
Brown antechinus *Antechinus stuartii*	Field	Most antechinus survive the fire. Post-fire torpor expression ~two-fold whereas activity is ~50% & largely nocturnal. Return to normal torpor/activity patterns 1 year post-fire.	Stawski *et al.* (2015a, 2016, 2017a)
Yellow-footed antechinus *Antechinus flavipes*	Field	Antechinus survive wildfire. Increased torpor expression post-fire and behavioural thermoregulation in blackened logs for energy conservation.	Matthews *et al.* (2017)
Yellow-footed antechinus *Antechinus flavipes*	Captive	Charcoal–ash substrate increases torpor duration ~2-fold in comparison to food restriction.	Stawski *et al.* (2017b)
Fat-tailed dunnart *Sminthopsis crassicaudata*	Captive	Smoke induces early arousals in torpid dunnarts and charcoal–ash reduces torpor use in this desert mammal.	Stawski *et al.* (2015b)
Eastern pygmy-possum *Cercartetus nanus*	Captive	Smoke induces arousal in torpid possums at *T*_a_ 15°C; at *T*_a_ 10°C response was reduced/slowed.	Nowack *et al.* (2016a)
Sugar glider *Petaurus breviceps*	Captive	Charcoal–ash substrate results in ~25% increase in torpor bout duration in comparison to food reduction alone.	Nowack *et al.* (2018)
Placentals			
Lesser long-eared bat *Nyctophilus geoffroyi*	Field	Bats increase activity and decrease torpor use after wildfire when insect abundance was high.	Doty *et al.* (2016a)
Long-eared bat *Nyctophilus gouldi*	Captive	Bats select black roosts over white, thermal biology affected by roost colour, more passive rewarming in black box.	Doty *et al.* (2016b)
Long-eared Bat *Nyctophilus gouldi*	Captive	Torpid bats respond to smoke within seconds, but exposure to cold slows response.	Doty *et al.* (2018)
Eastern red bat *Lasiurus borealis*	Field	Bats flushed by fire and ‘smoked’ from hibernaculum. Observed on ground still partially torpid attempting to fly or crawl.	Saugey *et al.* (1989) Moorman *et al.* (1999)
Eastern red bat *Lasiurus borealis*	Captive/field	One of 15 bats at *T*_a_ 5°C responded to sound of fire, all responded in 4–30 s when smoke and fire noise were combined and did arouse in 10–42 min.	Scesny (2006)
Eastern red bat *Lasiurus borealis*	Captive/field	Behavioural responses of bats negatively affected by *T*_a_ likely because they were torpid.	Layne (2009)
Big brown bat *Eptesicus fuscus* Eastern red bat *Lasiurus borealis* Eastern Pipistrelle *Perimyotis subflavus*	Field	Thinning of forest increases activity of bats more than burning.	Loeb and Waldrop (2008)
Meadow vole *Microtus pennsylvanicus*	Field	Voles flee to unburnt area during grassland fire or seek underground refuges. Low direct mortality from fire.	Geluso *et al.* (1986)
Mitchell’s hopping mouse *Notomys mitchelli* Sandy inland mouse *Pseudomys hermannsburgensis* House mouse *Mus musculus*	Field	Giving up densities lower in sheltered microhabitats in comparison to open microhabitats at recently burnt sites.	Doherty *et al.* (2015)
Golden-backed tree-rat *Mesembriomys macrurus*	Field	Select long unburnt rainforest over recently burnt savanna, but long unburnt savanna chosen least.	Hohnen *et al.* (2015)

There are numerous observations of bats flushing from roost sites in response to smoke and noise from a nearby fire (Dickinson *et al.*, 2009). For torpid long-eared bats (*N. gouldi*) the response to smoke, measured as an increase in respiration rate, was rapid and occurred within 1 s at *T*_a_ 21.4°C (*T*_b_ ~22.4°) and 36 s at *T*_a_ 11.9°C (*T*_b_ ~12.9°C). All bats rapidly (~8–15 min) rewarmed from torpor (Doty *et al.*, 2018). Eastern red bats, *L. borealis*, responded occasionally to fire noise at low *T*_a_, but when fire noise and smoke were combined all bats arose (Scesny, 2006). However, not all mammals detect fire cues and arouse in sufficient time to escape a fire. Two echidnas (*Tachyglossus aculeatus*) in torpor in the same hollow log were impacted by a management burn (Nowack *et al.*, 2016b). One arose and left the log, surviving the fire, while the other did not arouse from torpor. It died when the log burnt, without ever attaining a normothermic *T*_b_. Friend (1993) reports the discovery of charred brown and dusky antechinus (*A. stuartii and A. swainsonii*) carcases following a severe wildfire in heathland in south-east New South Wales.

After the cessation of smoke exposure, long-eared bats (*N. gouldi*) re-entered torpor, but they never returned to thermo-conforming, steady-state torpor prior to the end of the experimental day at low *T*_a_, although those at warmer *T*_a_ did (Doty *et al.*, 2018). Not returning to steady-state torpor at low *T*_a_ will increase energy expenditure, but presumably this cost is traded-off with the benefits of maintaining vigilance in the case of repeated smoke exposure. Even though torpid animals can respond to fire stimuli they may be slow in doing so, therefore torpid animals are at risk of not responding to fire cues quickly enough to survive. Planning of management burns should therefore consider daily and seasonal temperature conditions, and also ignition strategies to maximise smoke spread over the burn area ahead of a predictable, slowly-moving, low-intensity fire front. This will increase the likelihood of animals arousing from torpor and having the opportunity to escape the immediate effects of the fire.

## Post-fire responses of quadrupedal terrestrial mammals

New evidence suggests that torpor is used widely by terrestrial mammals to deal with fires or the scorched post-fire environment (Table 1). Echidnas, *T. aculeatus*, responded to fire by increasing the depth and duration of post-fire torpor bouts, compared to echidnas in unburnt areas (Nowack *et al.*, 2016b). Interestingly, echidnas reduced their daily activity but remained within their original home range, suggesting that animals can use the physiological option of torpor to minimise their energy needs sufficiently to remain in their original range, rather than moving into unburnt areas.

Forest-dwelling antechinus also increased torpor expression and duration and decreased daily activity in a post-fire environment (Stawski *et al.*, 2015a; Matthews *et al.*, 2017). The brown antechinus (*Antechinus stuartii*) increased torpor use and torpor duration after a hazard reduction burn by ~2-fold in comparison to the pre-fire controls and controls measured concurrently in an unburnt area nearby. At the same time activity decreased substantially (Stawski *et al.*, 2015a). Although torpor use by male *A. stuartii* was less than by females, the proportional change post-fire was similar for both sexes (Stawski *et al.*, 2016). The reduction in activity was mainly achieved by reducing daytime activity ranging from on average 2.7 to 4.7 h/d (males) and 2.4 to 3.3 h/d (females) to <0.4 h/d (both sexes) post-fire, likely to avoid exposure to predators in a habitat with little vegetation cover. Small terrestrial mammals are vulnerable to predation from both native and introduced predators after fires, due to reduced cover and influx of predators; for example, feral cats travel up to 12.5 km from their home range to a recently burnt area to hunt and birds of prey are also attracted to fires (Körtner *et al.*, 2007; Leahy *et al.*, 2016; McGregor *et al.*, 2016; Hovick *et al.*, 2017; Hradsky *et al.*, 2017). Nevertheless, antechinus remained in burned areas for weeks despite availability of unburned areas nearby and the population was still present one year after the fire, by which time the vegetation had recovered to a large extent and both torpor use and activity of antechinus had returned to pre-fire and control levels (Stawski *et al.*, 2017a). However, Recher *et al.* (2009) reported that a severe wildfire combined with drought lead to the disappearance of *A. stuartii*, along with another antechinus species (*A. swainsonii*) and a rodent (*Rattus fuscipes*) from a burnt area after a period of 18 months; they concluded that wildfire can have a catastrophic impact on small mammal populations in the longer-term, even if they persist in the shorter-term. Lunney *et al.* (1988) also observed disappearance of heterothermic antechinus *A. stuartii* and *A. swainsonii*, as well as the homeothermic *R. fuscipes*, after a forest fire, but the heterothermic dunnart (*Sminthopsis leucopus*) and house mouse (*Mus musculus*) persisted, and appeared to even benefit from a post-fire landscape. It is unclear if trapping-based studies are as likely to detect heterothermic animals that increase torpor use and reduce activity post-fire as radio-tracking studies, which may explain some discrepancy in results.

The yellow-footed antechinus, *A. flavipes*, another forest dweller, survived an extremely hot wildfire in south-eastern Australia that caused the mortality of many other mammals (Stawski *et al.*, 2014). Males used torpor on almost 80% of days, much more frequently than in a control site in a similar habitat where torpor occurred on less than 50% of days (Matthews *et al.*, 2017); in a female, torpor was used on almost 90% of days. After the fire, a male antechinus rested in blackened hollow logs during the daytime, likely because reduced canopy cover permitted increased exposure to solar radiation, resulting in the warming of logs and consequently a reduction in thermoregulatory energy expenditure (Matthews *et al.*, 2017). Basking during torpor has predominately been observed for desert marsupials that have access to high levels of solar radiation. This results in substantial energy savings due mostly to reduced costs of arousal and reduced thermoregulatory energy expenditure (Geiser *et al.*, 2004; Warnecke *et al.*, 2008).

Use of torpor in a post-fire environment by antechinus provides a plausible explanation as to why this genus is generally not as negatively impacted by fire as typically homeothermic species such as bush rats (*Rattus fuscipes*), which have high mortality rates (Thompson *et al.*, 1989; Recher *et al.*, 2009). Likely this is related to the required continued high intake of food in the rat that cannot be sustained, whereas the increased torpor use in antechinus permits a reduction in foraging and feeding, exposure to predators, and thus survival. This may also explain the observation that the majority of North American grassland small mammals that underwent a positive population response to fire were heterothermic, while most species that experienced a population decline were homeothermic (Kaufman *et al.*, 1990). There are several records of heterothermic deermice (*Peromyscus maniculatus*) remaining in burned areas post-fire, with good physical condition, although their use of torpor during this period was not reported (e.g. Crowner and Barrett, 1979; Zwolak and Foresman, 2008). However, in some cases fire can trigger canopy stored seed fall, and in these situations of increased post-fire resource abundance rodents such as house mice (*Mus musculus*) and *Pseudomys* spp. dominate the post-fire small mammal community (Friend, 1993). Interestingly, torpor has been documented for house mice and at least one *Pseudomys* spp. but it is not as pronounced as that observed for small dasyurid marsupials (Tomlinson *et al.*, 2007; Barker *et al.*, 2012).

Although much of the increase in post-fire torpor use is likely a consequence of a long-term decrease in food availability and lack of cover, recent data for captive mammals indicate that the presence of charcoal–ash substrate and smoke enhances mammalian torpor use beyond that induced by food restriction alone. This suggests that these post-fire cues signal a period of imminent food shortage and increased risk (Stawski *et al.*, 2017b; Table 1). For yellow-footed antechinus, smoke exposure and a charcoal–ash substrate after withdrawal of food resulted in an almost 2-fold increase in daily torpor duration and a more substantial *T*_b_ reduction in comparison to food restriction alone or food restriction with smoke exposure (Stawski *et al.*, 2017b). For arboreal sugar gliders, *Petaurus breviceps*, food reduction and a charcoal–ash substrate resulted in a ~25% prolongation of torpor bouts in comparison to food restriction alone (Nowack *et al.*, 2018).

Desert-dwelling dunnarts (*S. crassicaudata*) responded differently to post-fire cues compared with small forest-dwelling mammals. When provided with food and exposed to a charcoal/ash substrate, minimum *T*_b_ increased and activity decreased. When food was withheld, torpor expression on a charcoal/ash substrate was similar to the control substrate (Stawski *et al.*, 2015b). However, the incidence of daily torpor use by dunnarts is very high and reaches 100% in the wild (Warnecke *et al.*, 2008) and therefore cannot increase further, in comparison to the on average 50% torpor expression for free-ranging antechinus (Matthews *et al.*, 2017).

## The effects of fire on heterothermic bats

Bats have an advantage over small terrestrial quadrupedal mammals because flight provides an enormous increase in mobility and low cost of locomotion (Tucker, 1975; Withers *et al.*, 2016). Volant bats therefore may be able to escape threats such as fire more easily than other small terrestrial mammals, which are restricted to locating refugia in trees or burrows by moving at rather low speed on the substrate. However, with over 1300 species of bats worldwide, responses to fire are likely highly variable and dependent on niche occupation and life history of the species in question. Fire can be beneficial to bats because it can create or widen tree hollows (Lunney *et al.*, 1988), but may also destroy hollows (Parnaby *et al.*, 2010, 2011). A reduction in spatial complexity and clutter following fire also permits less manoeuvrable bats (generally larger-bodied bats) to access habitats which previously were too spatially complex for foraging and roosting (Betts, 2009; Inkster-Draper *et al.*, 2013). Generally bats have an overall positive or neutral response to fire, with either no change or an increase in activity following management burns (Milne *et al.*, 2005; Lloyd *et al.*, 2006; Loeb and Waldrop, 2008; Betts, 2009; Smith and Gehrt, 2010; Johnson *et al.*, 2011; Armitage and Ober, 2012; Inkster-Draper *et al.*, 2013; Starbuck *et al.*, 2015; Cox *et al.*, 2016; Silvis *et al.*, 2016; Lacki *et al.*, 2017) and wildfires (Malison and Baxter, 2010; O’Shea *et al.*, 2011; Homan, 2012; Buchalski *et al.*, 2013; Doty *et al.*, 2016a; Law *et al.*, 2018). There are exceptions for some species, such as the southern myotis (*Myotis macropus*), an Australian fishing bat, and the long-eared myotis (*Myotis evotis*), a North American crevice-roosting bat, both of which actively avoid fire-burnt habitat (Lloyd *et al.*, 2006; Snider *et al.*, 2013). Some larger-bodied bats such as the Eastern red bat (*Lasiurus borealis*) are less active in burnt areas (Loeb and Waldrop, 2008; Silvis *et al.*, 2016), which may be related to post-fire insect abundance or the roosting ecology of the species. Eastern red bats roost under leaf litter and are especially susceptible to fires (Scesny, 2006; Layne, 2009; Perry and McDaniel, 2015).

Data concerning the physiological responses of bats to fire are scarce (Doty *et al.*, 2018). The lesser long-eared bat (*Nyctophilus geoffroyi*, 6–8 g), a common Australian insectivorous bat, modified patterns of torpor use following an extensive wildfire (Doty *et al.*, 2016a). Although this bat used torpor on all measurement days, mean torpor bout duration 4 months post-fire was ~12 h in comparison to ~24 h 2 years later. The species was also active or normothermic more often and for longer periods 4 months after the wildfire compared to 2 years later. The reasons for this may be due to the 20-fold greater insect abundance for months following the wildfire, encouraging the bats to forage for longer periods of time. Raptors and insectivorous and granivorous passerine birds also increase foraging in recently burnt areas (Dean, 1987; Doty *et al.*, 2015; Hovick *et al.*, 2017). Additionally, the landscape was largely denuded and uncluttered 4 months following the fire, allowing for easier foraging by bats and more solar penetration to roost sites compared to two 2 years after the fire (Doty *et al.*, 2016a). But even under these apparently favourable conditions, bats still were torpid for about half the time (Doty *et al.*, 2016a) emphasising the importance of energy conservation for small insectivorous bats.

Increased solar penetration to roost sites is physiologically beneficial for many species of insectivorous bat, allowing for passive rewarming, or the close tracking of *T*_b_ with *T*_a_, over a greater range of *T*_a_ without the need of a substantial increase in metabolism (Vaughan and O’Shea, 1976; Hamilton and Barclay, 1994; Chruszcz and Barclay, 2002; Turbill *et al.*, 2003; Geiser *et al.*, 2004; Turbill, 2006; Bondarenco *et al.*, 2014; Doty *et al.*, 2016b). Post-fire habitat is often comparatively less spatially complex than unmanaged or unburnt landscapes, facilitating increased solar exposure to trees. Although some bats reduce energy expenditure during torpor as much as 99% (Ruf and Geiser, 2015), periodic arousal to normothermia during hibernation can account for as much as 83–95% of the total energy expenditure of small mammals (Wang, 1978; Geiser, 2007). Passive or partially passive rewarming by bats can reduce energy expenditure associated with arousal from torpor by as much as 53%, by decreasing the temperature range over which active arousal is required, and may also reduce the associated cardiac demands (Turbill and Geiser, 2008; Currie *et al.*, 2015; Doty *et al.*, 2016a). The primary purpose of periodic arousals from torpor is not well understood. But they may provide an opportunity for sleep and neural re-generation, may remove accumulated metabolic by-products, stimulate immune system function or facilitate maintenance of water balance (Pengelley and Fisher, 1961; Geiser *et al.*, 1990; Withers and Cooper, 2008). Whatever the purpose, it seems that periodic return to a *T*_b_ that approximates normothermia is necessary for most hibernators, including bats (Withers *et al.*, 2016), and midday arousals, facilitated by passive rewarming, are an energetically beneficial means for small mammals to achieve and maintain normothermia at a reduced energetic cost (Mzilikazi *et al.*, 2002; Geiser *et al.*, 2004; Mzilikazi and Lovegrove, 2004; McKechnie and Mzilikazi, 2011; Dausmann, 2014). Tree-roosting bats will often choose the sunny sides of roosts, thermally unstable roosts, darker roosts, or roosts located in stands with less vegetative complexity and crown density and greater canopy gaps, which permits more solar exposure (Callahan *et al.*, 1997; Turbill *et al.*, 2003; Turbill, 2006; Doty *et al.*, 2016b; O’Keefe and Loeb, 2017). Following both wild and management fires, bats often choose roost sites with greater solar exposure or choose burnt landscapes over unburnt sites (Boyles and Aubrey, 2006; Johnson *et al.*, 2009, 2010; O’Keefe and Loeb, 2017). Fire management of landscapes may therefore be beneficial for the creation or maintenance of physiologically favourable roosts.

The preference of bats for dark-coloured roosts has been demonstrated by studies on captive bats. Black roosts, which reach higher internal *T*_a_s are usually preferred over white boxes as long as *T*_a_ is not too high (Lourenço and Palmeirim, 2004; Doty *et al.*, 2016b), presumably as black boxes permit more extensive passive rewarming as well as allowing them to remain normothermic for long periods with low thermoregulatory energy expenditure. Roosts blackened by fire likely have similar thermal characteristics (Doty *et al.*, 2016b).

In the northern hemisphere, where many bat species hibernate over winter in caves or mines rather than in trees, poorly ventilated caves could be problematic in the event of a fire. Unfortunately, there are few data on the effects of smoke and fire on cave-roosting bats, with one study reporting that bats did not respond to smoke intrusion from a winter management burn (Caviness, 2003). Some North American bats, particularly lasiurine species such as eastern red bats, roost in leaf litter during colder months. As mentioned above, red bats have been observed flushing from leaf litter in response to winter management burns (Saugey *et al.*, 1989; Moorman *et al.*, 1999). Tree-roosting northern long-eared bats (*Myotis septentrionalis*) also fly away from the fires, but do not appear to leave their general home range (Dickinson *et al.*, 2009). Perry and McDaniel (2015) determined that high ground temperatures of up to 717°C during management burning resulted in only 5% of study plots being survivable for litter-roosting bats, indicating that management burns conducted during winter are particularly dangerous for torpid bats on the ground that may not have sufficient time to rewarm and escape. Carbon monoxide levels are also dangerous for bats roosting on or close to the ground, but are less fatal with greater roost height and increased wind (Dickinson *et al.*, 2010). In contrast, heat injury to bats may occur at flame heights similar to that which causes foliage necrosis (Dickinson *et al.*, 2010). Overall, management burns conducted at warmer temperatures, if the fire can be controlled and occurs at low intensity, may result in greater survivability for torpid bats, as bats take less time to rewarm from torpor at warmer *T*_a_ (Dunbar and Tomasi, 2006), and winter burns during cold weather may be particularly threatening.

## The implications of behavioural physiology for understanding mammalian fire ecology

Our summary provides further evidence that daily torpor and hibernation provide heterothermic mammals with an adaptive advantage over homeothermic species in changing environments, due to flexible thermal energetics. Heterothermic species do not only use torpor to survive seasonal energetic and thermal challenges, but also to endure the consequences of unpredictable energy bottlenecks or natural disasters and overall this results in lower risk of extinction (Geiser and Turbill, 2009; Turbill *et al.*, 2011; Hanna and Cardillo, 2014). As human-induced environmental change precedes at an unpreceded rate into the Anthropocene, opportunistic heterothermic species may be best positioned to withstand the rapid and major environmental challenges facing mammals into the future.

Ecological studies, especially those using a trap and release approach, often assume that pre- and post-fire trapping are directly comparable and reflect mammal populations in the same way. This approach cannot reveal the basic behaviour and physiology crucial to understanding the mechanisms of mammalian fire ecology. Moreover, many of these studies have produced ambiguous results and often assume that any observed changes reflect actual changes in diversity and abundance. As our summary shows, many small terrestrial mammals increase torpor use and reduce activity in a post-fire landscape, which may substantially affect trap success. Thus, inferences concerning diversity and abundance in post-fire environments based on trapping studies alone, without data on the behaviour and function of mammals, may not realistically represent changes in populations due to fire and therefore may not be suitable for generating reliable predictive models that have a good probability for improving animal conservation.

Based on current knowledge of mammalian fire responses reviewed here, we suggest that further research is required to assess the functional responses of mammals, especially small terrestrial mammals, to fire, particularly wildfire. Larger mammals and bats may have an overall net energetic benefit from fires. For small forest-dwelling mammals, heterothermia appears to provide for increased resilience to fire, at least in the short to medium-term, due to reduction in energy requirements and reduced exposure to predation. However, longer-term effects are not well understood, and the immediate threat of fire can have varied impacts for animals in torpor at the time of the fire. More research is required to understand fire responses of small heterothermic mammals from arid habitats, and the potential impacts of burns in their environment. Based on current knowledge, we recommend that fire management protocols consider the extent of heterothermic species comprising small mammal populations, and conduct management burns accordingly. Survival and retention of small heterothermic terrestrial mammals likely will be improved if very cold periods when deep torpor is common are avoided and when control burns are moving at a slow pace at low intensity and create plenty of smoke to provide early warning of the imminent fire.

## Abbreviations

*T*_a_: ambient temperature
*T*_b_: body temperature
